# Risk Factors for Acute Kidney Injury in Critically Ill Neonates: A Systematic Review and Meta-Analysis

**DOI:** 10.3389/fped.2021.666507

**Published:** 2021-07-14

**Authors:** Qian Hu, Shao-Jun Li, Qian-Ling Chen, Han Chen, Qiu Li, Mo Wang

**Affiliations:** ^1^Department of Nephrology, Ministry of Education Key Laboratory of Child Development and Disorders, National Clinical Research Center for Child Health and Disorders, China International Science and Technology Cooperation Base of Child Development and Critical Disorders, Children's Hospital of Chongqing Medical University, Chongqing, China; ^2^Department of Emergency Department, Children's Hospital of Chongqing Medical University, Chongqing, China; ^3^Department of Nephrology and Rheumatism, Hainan Women and Children's Medical Center, Haikou, China

**Keywords:** neonates, acute kidney injury, risk factors, systematic review, meta-analysis

## Abstract

**Background and Objective:** Acute kidney injury (AKI) is recognized as an independent risk factor for mortality and long-term poor prognosis in neonates. The objective of the study was to identify the risk factors for AKI in critically ill neonates to provide an important basis for follow-up research studies and early prevention.

**Methods:** The PubMed, Embase, Web of Science, Cochrane Library, China National Knowledge Infrastructure, WanFang Med, SinoMed, and VIP Data were searched for studies of risk factors in critically ill neonates. Studies published from the initiation of the database to November 19, 2020, were included. The quality of studies was assessed by the Newcastle-Ottawa Scale and the Agency for Healthcare Research and Quality (AHRQ) checklist. The meta-analysis was conducted with Stata 15 and drafted according to the guidelines of the Preferred Reporting Items for Systematic reviews and Meta-Analyses (PRISMA) statement.

**Results:** Seventeen studies (five cohort studies, ten case-control studies, and two cross-sectional studies) were included in meta-analysis, with 1,627 cases in the case group and 5,220 cases in the control group. The incidence of AKI fluctuated from 8.4 to 63.3%. Fifteen risk factors were included, nine of which were significantly associated with an increased risk of AKI in critically ill neonates: gestational age [standardized mean difference (SMD) = −0.31, 95%CI = (−0.51, −0.12), *P* = 0.002], birthweight [SMD = −0.37, 95%CI = (−0.67, −0.07), *P* = 0.015], 1-min Apgar score [SMD = −0.61, 95%CI = (−0.78, −0.43), *P* = 0.000], 5-min Apgar score [SMD = −0.71, 95%CI = (−1.00, −0.41), *P* = 0.000], congenital heart disease (CHD) [odds ratio (OR) = 2.94, 95%CI = (2.08, 4.15), *P* = 0.000], hyperbilirubinemia [OR = 2.26, 95%CI = (1.40, 3.65), *P* = 0.001], necrotizing enterocolitis (NEC) [OR = 6.32, 95%CI = (2.98, 13.42), *P* = 0.000], sepsis [OR = 2.21, 95%CI = (1.25, 3.89), *P* = 0.006], and mechanical ventilation [OR = 2.37, 95%CI = (1.50, 3.75), *P* = 0.000]. Six of them were not significantly associated with AKI in critically ill neonates: age [SMD = −0.25, 95%CI = (−0.54, 0.04), *P* = 0.095], male sex [OR = 1.10, 95%CI =(0.97, 1.24), *P* = 0.147], prematurity [OR = 0.90, 95%CI(0.52, 1.56), *P* = 0.716], cesarean section [OR = 1.52, 95%CI(0.77, 3.01), *P* = 0.234], prenatal hemorrhage [OR = 1.41, 95%CI = (0.86, 2.33), *P* = 0.171], and vancomycin [OR = 1.16, 95%CI = (0.71, 1.89), *P* = 0.555].

**Conclusions:** This meta-analysis provides a preliminary exploration of risk factors in critically ill neonatal AKI, which may be useful for the prediction of AKI.

**Systematic Review Registration:** PROSPERO (CRD42020188032).

## Introduction

Acute kidney injury (AKI) is characterized by an abrupt decrease in kidney function, which is significantly associated with increased mortality in neonates (1, 2). Due to a number of features of neonatal renal physiology including tubular immaturity and low renal blood flow, the incidence of neonatal AKI has been reported to be high (3). This increased risk for AKI makes early identification of potential risk factors for AKI in neonates important so that they can benefit from potential preventive strategies. Many studies on the risk factors of AKI in critically ill neonates were published, but there are differences between their results (2, 4–6). Though several studies have clarified some risk factors, there is a lack of meta-analysis evaluating these risk factors associated with the occurrence of AKI in critically ill neonates (3, 7–9).

This study was designed to perform a meta-analysis to identify risk factors associated with AKI in critically ill neonates. It may be helpful for the prediction of AKI in critically ill neonates.

## Methods

This meta-analysis was reported in accordance with the guidelines of the Preferred Reporting Items for Systematic Reviews and Meta-analyses (PRISMA) statement (10). The protocol for this systematic review was registered on the International Prospective Register of Systematic Reviews (PROSPERO) (registration number: CRD42020188032). This meta-analysis was conducted on the neonates admitted to the neonatal intensive care unit. Risk factors to be investigated included gestational age, birthweight, 1-min Apgar score, 5-min Apgar score, congenital heart disease (CHD), hyperbilirubinemia, necrotizing enterocolitis (NEC), mechanical ventilation, age, male sex, prematurity, Cesarean section, prenatal hemorrhage, sepsis, and vancomycin. We included cohort studies, case-control studies, and cross-sectional studies that investigated AKI as an outcome.

### Data Sources and Searches

We conducted an electronic search of PubMed, Embase, Web of Science, Cochrane Library, China National Knowledge Infrastructure, WanFang Med, VIP Data, and SinoMed with the keywords including “neonates,” “acute kidney injury,” “risk factors,” and “risk.” Retrieval time was from inception to November 19, 2020. Search terms and Boolean operators included in the search strategies of PubMed and Embase are presented in online Supplementary Material 1.

### Study Selection

Study selection was independently conducted by QH and SJL, with any discrepancies resolved by MW. Inclusion criteria were as follows: (1) Patients are neonates admitted to the neonatal intensive care unit; (2) the risk factors for AKI in neonates are reported; and (3) the definition of AKI is clear, such as Kidney Disease: Improving Global Outcomes (KDIGO) definition, Acute Kidney Injury Network (AKIN) definition, or arbitrary definition (1, 11, 12). Exclusion criteria were as follows: (1) reviews, case reports, nonclinical studies, and the studies inconsistent with the purpose of evaluation; (2) full data cannot be provided; (3) repetitive reports; and (4) non-English or non-Chinese literature studies.

### Data Collection and Extraction

Data were independently extracted by QH and S-JL, with any discrepancies resolved by MW. Data collected included the characteristics of the studies, the demographic characteristics of the patients, accompanying diseases, and therapeutic measures. When full data cannot be obtained from the study, we tried to contact the corresponding author to obtain all the data.

### Quality Assessment

Quality assessment was independently conducted by QH and S-JL, with any discrepancies resolved by MW. The quality of cohort and case-control studies was assessed using the Newcastle-Ottawa Scale (NOS), which was widely used in the quality assessment of case-control and cohort studies (13, 14). The NOS conducts a comprehensive evaluation from three aspects of the study: selection, comparability, and outcome (cohort studies) or exposure (case-control studies). A study can be awarded a maximum of one point for each numbered item within the selection and exposure categories. A maximum of two points can be given for comparability. The quality of the study was assessed as follows: low quality = 0–3; moderate quality = 4–6; and high quality = 7–9 (15). The quality of cross-sectional studies was assessed by the 11-item checklist recommended by the Agency for Healthcare Research and Quality (AHRQ), which included the definition of information source, inclusion and exclusion criteria, time period and continuity for identifying patients, blinding of personnel, assessments for quality assurance, confounding and missing data, and response rates and completeness of patients. An item would be scored “0” if it was answered “UNCLEAR” or “NO”; for the answer of “YES,” the item would get a score of “1.” Quality of the study was assessed as follows: low quality = 0–3; moderate quality = 4–7; and high quality = 8–11 (16, 17).

### Statistical Analysis

Effect sizes have been reported in odds ratio (OR) for dichotomous data and standardized mean difference (SMD) for continuous outcomes. Raw data of continuous variables were converted into mean and standardized difference (SD) wherever possible (18). Pooled effect estimates were reported with 95% CIs. Heterogeneity was tested using the *I*^2^ test, with *I*^2^ > 50%, or *p*-value < 0.1 was considered significant. If there was significant heterogeneity, a random-effects model was used or else a fixed-effects model. Statistical significance was defined as a two-tailed *p*-value < 0.05. Sensitivity analyses were conducted on each risk factor by removing each individual study from the overall analysis. Subgroup analyses were performed on the risk factors with significant heterogeneity, which were based on the definition of AKI (KDIGO or non-KDIGO) and research method (cohort or non-cohort study) (1). Publication bias was estimated *via* Egger's test, and a *p* > 0.05 was considered non-significant publication bias. If there was publication bias, the non-parametric clipping was used to evaluate the impact of publication bias on the results. All statistical analyses were performed using Stata 15.0 software (19).

## Results

### Characteristics of Included Studies

**Figure 1:**
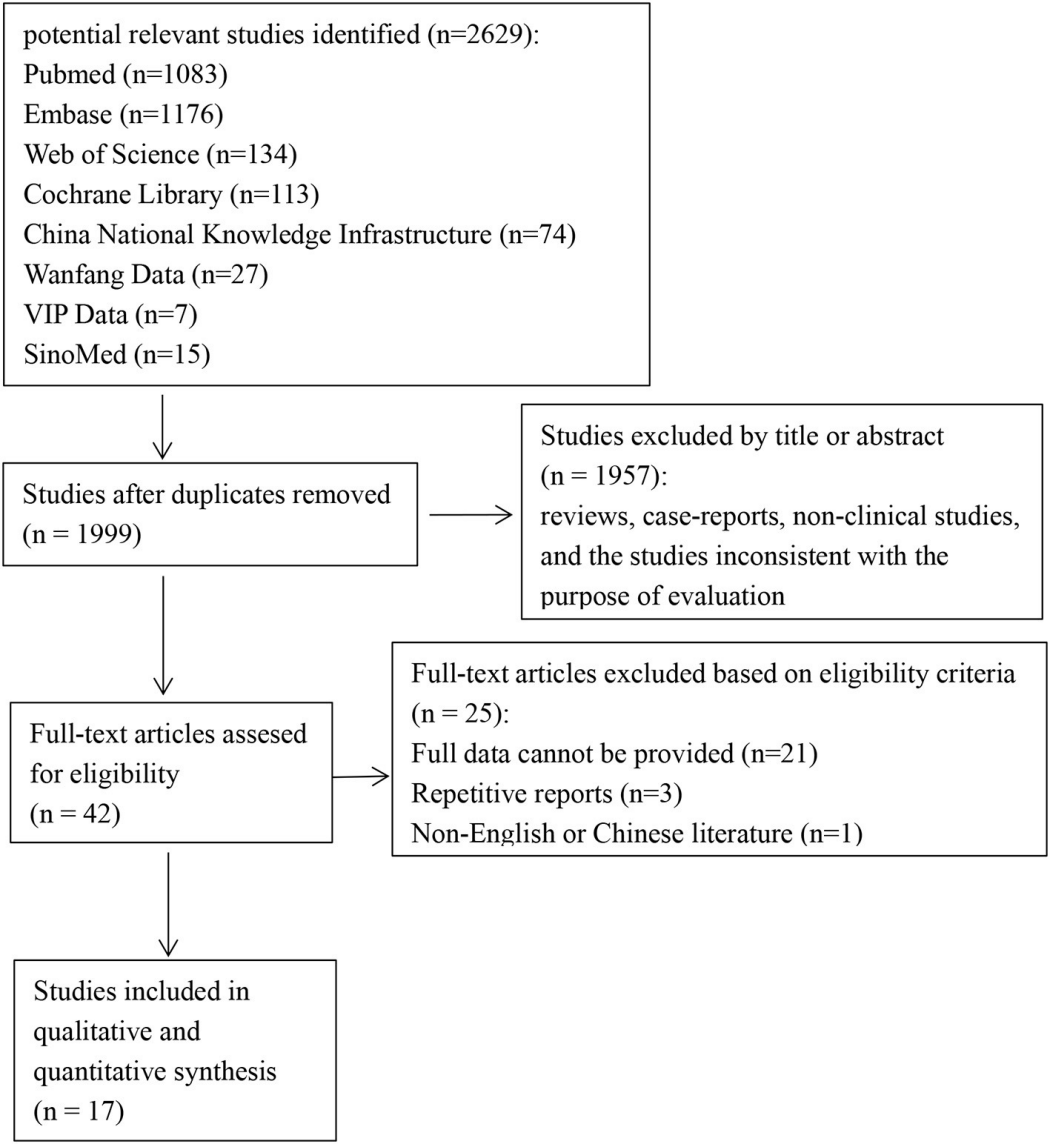
Flowchart of the selection process for eligible studies [the preferred reporting items for systematic reviews and meta-analyses (PRISMA) 2009 flow diagram].

Initial screening identified 2,629 publications (Figure 1). Finally, only 17 studies satisfied our inclusion criteria and were involved in the meta-analysis (2, 4–6, 12, 20–31), including five cohort studies, ten case-control studies, and two cross-sectional studies (Table 1). Of these, nine studies employed the KDIGO definition or the KDIGO definition modified for neonates (mKDIGO), six studies employed arbitrary definitions, and two studies employed the AKIN definition. The 17 studies included in qualitative analysis contributed to 1,627 cases and 5,220 controls. The incidence of neonatal AKI fluctuates between 8.4 and 63.3%.

**Table 1:** Basic characteristics of included studies.

**References**	**Country**	**AKI (*n*)**	**Non-AKI (*n*)**	**Incidence rate (%)**	**Definition of AKI**	**Research method**
Fonseca et al. (12)	Mexico	47	53	47.0	arbitrary	case-control
Türker et al. (22)	Turkey	78	475	14.1	arbitrary	case-control
Bolat et al. (23)	Turkey	168	1,824	8.4	arbitrary	case-control
El-Badawy et al. (27)	Egypt	41	59	41	arbitrary	cohort
Kriplani et al. (28)	America	28	52	35	mKDIGO	case-control
Zhang et al. (29)	China	75	140	34.8	KDIGO	case-control
Bansal et al. (4)	India	74	100	-	arbitrary	case-control
Jetton et al. (2)	multicenter	605	1,417	29.9	mKDIGO	cohort
Ghobrial et al. (24)	Egypt	30	60	-	arbitrary	case-control
Shalaby et al. (5)	Saudi Arabia	120	94	56.1	mKDIGO	cohort
Gong et al. (21)	China	35	101	25.7	AKIN	cohort
Liu et al. (25)	China	32	212	13.1	AKIN	case-control
Lei et al. (26)	China	76	44	63.3	mKDIGO	case-control
Mazaheri et al. (30)	Iran	20	186	9.7	mKDIGO	cross-sectional
Mwamanenge et al. (6)	Tanzania	119	259	31.5	KDIGO	cross-sectional
Hamsa et al. (20)	India	49	114	30.0	mKDIGO	cohort
El-sadek et al. (31)	multicenter	30	30	-	mKDIGO	case-control

*AKI, acute kidney injury; KDIGO, kidney disease: improving global outcomes definition; mKDIGO, kidney disease: improving global outcomes definition modified for neonates; AKIN, acute kidney injury network definition*.

### Quality Assessment

Based on the NOS quality assessment and AHRQ checklist, 14 studies were classified as high quality and three studies as moderate quality (Tables 2–4). The comparability scores of the two medium-quality case-control studies are both zero. In the cohort and case-control studies, the controls were not community based.

**Table 2:** Newcastle-Ottawa Scale (cohort) for five studies^a^ included in this meta-analysis.

**Item**		**I**	**II**	**III**	**IV**	**V**
Representativeness of the exposed cohort	a) truly representative of the average __(describe) in the community#; b) somewhat representative of the average __in the community#; c) selected group of users, e.g., nurses, volunteers; d) no description of the derivation of the cohort	1	1	1	1	1
Selection of the nonexposed cohort	a) drawn from the same community as the exposed cohort#; b) drawn from a different source; c) no description of the derivation of the nonexposed cohort	0	0	0	0	0
Ascertainment of exposure	a)secure record (e.g., surgical records)#; b) structured interview#; c) written self-report; d) no description	1	1	1	1	1
Demonstration that outcome of interest was not present at start of study	a) yes#; b) no	1	1	1	1	1
Comparability of cohorts on the basis of the design or analysis	a) study controls for __ (select the most important factor)#; b) study controls for any additional factor# (These criteria could be modified to indicate specific control for a second important factor.)	1	1	1	1	1
Assessment of outcome	a) independent blind assessment#; b) record linkage# c) self-report; d) no description	1	1	1	1	1
Was follow-up long enough for outcomes to occur	a) yes (select an adequate follow-up period for outcome of interest)#; b) no	1	1	1	1	1
Adequacy of follow-up of cohorts	a) complete follow-up - all subjects accounted for#; b) subjects lost to follow-up unlikely to introduce bias - small number lost - > __ % (select an adequate %) follow-up, or description provided of those lost)#; c) follow-up rate < ___% (select an adequate %) and no description of those lost; d) no statement	1	1	1	1	1
Score		7	7	7	7	7

a *Studies: I = (2); II = (5); III = (20); IV = (21); V = (27)*.

# *One point*.

**Table 3:** Newcastle-Ottawa Scale (case-control) for ten studies^a^ included in this meta-analysis.

**Item**		**I**	**II**	**III**	**IV**	**V**	**VI**	**VII**	**VIII**	**IX**	**X**
Was the case definition adequate	a. Yes, with independent validation^∧^; b. yes, e.g., record linkage or based on self-reports; c. no description	1	1	1	1	1	1	1	1	1	1
Representativeness of the cases	a. Consecutive or obviously representative series of cases^∧^; b. potential for selection biases or not stated	1	1	1	1	1	1	1	1	1	1
Selection of controls	a. Community controls^∧^; b. hospital controls; c. no description	0	0	0	0	0	0	0	0	0	0
Definition of controls	a. No history of disease (endpoint)^∧^; b. no description of source	1	1	1	1	1	1	1	1	1	1
Comparability	a. Study controls for_ _ _ _(selecting the most important factor)^∧^; b. study controls for any additional factor^∧^ (These criteria could be modified to indicate specific control for a second important factor.)	0	1	1	0	1	1	1	1	1	2
Ascertainment of exposure	a. secure records (e.g., surgical records)^∧^; b. structured interview blinded to case/control status^∧^; c. Interview not blinded to case/control status; d. written self-report or medical record only; e. no description	1	1	1	1	1	1	1	1	1	1
Same method of ascertainment for cases and controls	a. yes^∧^; b. no	1	1	1	1	1	1	1	1	1	1
Non-Response rate	a. Same rate for both groups^∧^; b. non-respondents described; c. rate different and no designation	1	1	1	1	1	1	1	1	1	1
Total score		6	7	7	6	7	7	7	7	7	8

a *Studies: I = (12); II = (22); III = (23); IV = (4); V = (24); VI = (25); VII = (26); VIII = (28); IX = (29); and X = (31)*.

∧ *One point*.

**Table 4:** Agency for healthcare research and quality (AHRQ) checklist (cross-sectional) for 2 studies^a^ included in this meta-analysis.

**Item**	**I**	**II**
1) Define the source of information (survey, record review)	1	1
2) List inclusion and exclusion criteria for exposed and unexposed subjects (cases and controls) or refer to previous publications.	1	1
3) Indicate time period used for identifying patients.	1	1
4) Indicate whether or not subjects were consecutive if not population-based.	1	1
5) Indicate if evaluators of subjective components of study were masked to other aspects of the status of the participants.	1	1
6) Describe any assessments undertaken for quality assurance purposes (e.g., test/retest of primary outcome measurements).	0	1
7) Explain any patient exclusions from analysis.	1	1
8) Describe how confounding was assessed and/or controlled.	0	1
9) If applicable, explain how missing data were handled in the analysis.	0	0
10) Summarize patient response rates and completeness of data collection.	0	0
11) Clarify what follow-up, if any, was expected and the percentage of patients for which incomplete data or follow-up was obtained.	0	1
Total score	6	9

a *Studies: I = (30); II = (6)*.

### Results of Meta-Analysis

The analyses of risk factors are shown in Table 5. The heterogeneities of age, gestational age, birthweight, Cesarean section, 1-min Apgar score, 5-min Apgar score, prematurity, sepsis, and mechanical ventilation were significant, which, in turn, used the random-effects models. As for male sex, prenatal hemorrhage, CHD, hyperbilirubinemia, NEC, and vancomycin, the heterogeneities were not significant, so fixed-effects models were used. Compared to the non-AKI group, the AKI group had lower values of gestational age [SMD = −0.31, 95%CI = (−0.51, −0.12), *P* = 0.002] (Figure 2), birthweight [SMD = −0.37, 95%CI = (−0.67, −0.07), *P* = 0.015], 1-min Apgar score [SMD = −0.61, 95%CI = (−0.78, −0.43), *P* = 0.000], and 5-min Apgar score [SMD = −0.71, 95%CI = (−1.00, −0.41), *P* = 0.000]. As compared to the non-AKI group, the AKI group had higher incidences of comorbidities such as CHD [OR = 2.94, 95% CI = (2.08, 4.15), *P* = 0.000], hyperbilirubinemia [OR = 2.26, 95%CI = (1.40, 3.65), *P* = 0.001], NEC [OR = 6.32, 95%CI = (2.98, 13.42), *P* = 0.000], and sepsis [OR = 2.21, 95%CI = (1.25, 3.89), P = 0.006]. Compared to the non-AKI group, the AKI group was more likely to use mechanical ventilation [OR = 2.37, 95%CI = (1.50, 3.75), *P* = 0.000]. Age [SMD = −0.25, 95%CI = (−0.54, 0.04), *P* = 0.095], male sex [OR = 1.10, 95%CI = (0.97, 1.24), *P* = 0.147], prematurity [OR = 0.90, 95%CI (0.52, 1.56), *P* = 0.716], Cesarean section [OR = 1.52, 95%CI (0.77, 3.01), *P* = 0.234], prenatal hemorrhage [OR = 1.41, 95% CI = (0.86, 2.33), *P* = 0.171], and vancomycin [OR = 1.16, 95% CI = (0.71, 1.89), *P* = 0.555] were not significantly associated with AKI in critically ill neonates.

**Table 5:** Results of meta-analysis.

**Risk factors**	**Number of studies**	**Net change (95% CI)**	***P***	**Heterogeneity**		**Analysis model**	**Egger's test**
				***I*****^2^ (%)**	***P***		
Age	5	−0.25 (−0.54, 0.04)#	0.095	61.3	0.035	Random	*P* = 0.341
Male sex	15	1.10 (0.97, 1.24)^∧^	0.147	18.2	0.25	Fixed	*P* = 0.393
Gestational age	10	−0.31 (−0.51, −0.12)#	0.002	67.8	0.001	Random	*P* = 0.511
Prematurity	6	0.90 (0.52, 1.56)^∧^	0.716	76.4	0.001	Random	*P* = 0.923
Birthweight	8	−0.37 (−0.67, −0.07)#	0.015	84.1	0.000	Random	*P* = 0.800
Cesarean section	3	1.52 (0.76, 3.01)^∧^	0.234	74.5	0.020	Random	
Apgar 1	10	−0.61 (−0.78, −0.43)#	0.000	66.2	0.002	Random	*P* = 0.020
Apgar 5	10	−0.71 (−1.00, −0.41)#	0.000	91.3	0.000	Random	*P* = 0.140
Antepartum hemorrhage	2	1.41 (0.86, 2.33)^∧^	0.171	0.0	0.622	Fixed	
Sepsis	11	2.21 (1.25, 3.89)^∧^	0.006	89.5	0.000	Random	*P* = 0.003
Congenital heart disease	6	2.94 (2.08, 4.15)^∧^	0.000	0.0	0.558	Fixed	*P* = 0.426
Hyperbilirubinemia	2	2.26 (1.40, 3.65)^∧^	0.001	0.0	0.726	Fixed	
Necrotizing enterocolitis	4	6.32 (2.98, 13.42)^∧^	0.000	0.0	0.975	Fixed	*P* = 0.385
Mechanical ventilation	8	2.37 (1.50, 3.75)^∧^	0.000	66.5	0.004	Random	*P* = 0.392
Vancomycin	2	1.16 (0.71, 1.89)^∧^	0.555	0	0.700	Fixed	

∧ *Odds ratio (OR) and 95% CI;*

# *standardized mean difference (SMD) and 95% CI*.

**Figure 2:**
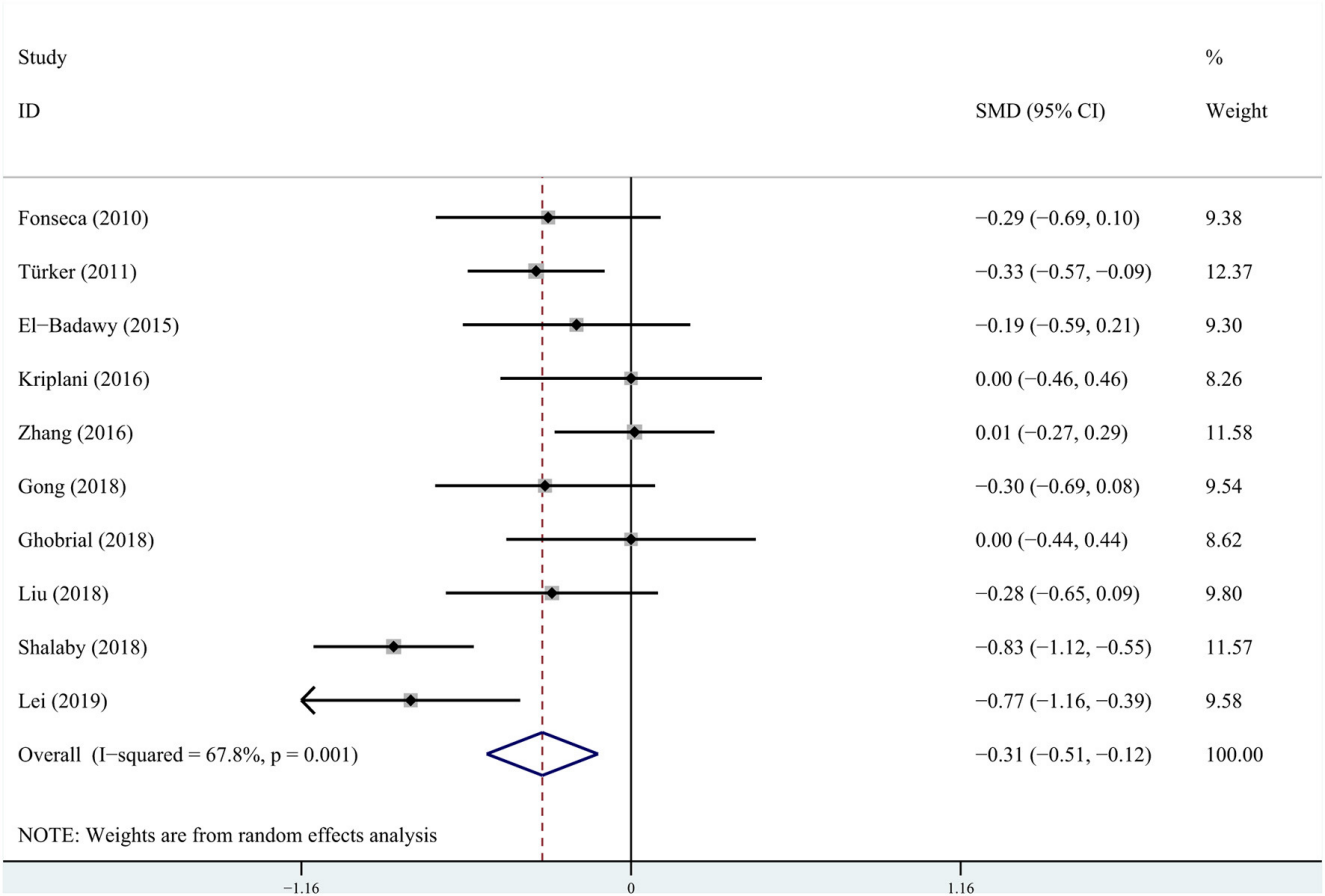
Pooled standardized mean difference (SMD) for gestational age from random-effects meta-analysis.

### Sensitivity and Subgroup Analyses

**Figure 3:**
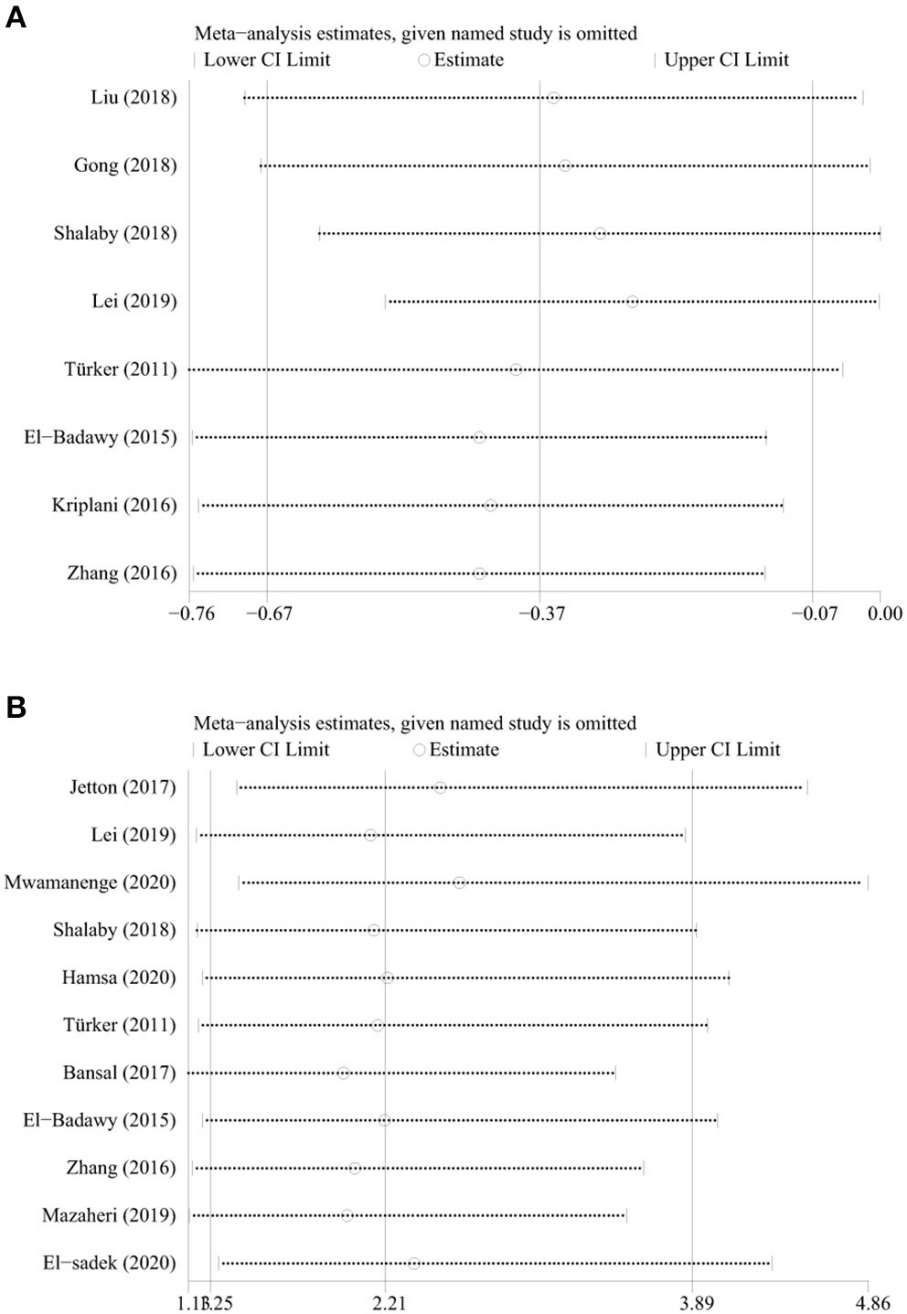
**(A)** Sensitivity analysis for birthweight; **(B)** sensitivity analysis for sepsis.

The sensitivity analyses for each risk factor showed that no individual study significantly altered the results. The results for birthweight and sepsis were shown in Figure 3. Subgroup analyses based on the research method showed that both cohort and non-cohort studies had similar results except for sepsis (Table 6). Because the studies included in the age and Cesarean section were all non-cohort studies, subgroup analyses based on the research method were not conducted. Subgroup analyses based on the definition of AKI revealed that both studies of KDIGO definition and non-KDIGO definition had similar results except for age, birthweight, and sepsis (Table 7). Because the studies included in the Cesarean section were all non-KDIGO defined, subgroup analysis based on the definition of AKI was not conducted.

**Table 6:** Subgroup analyses based on the research method.

**Risk factors**	**Research methods**	**Number of trials**	**Net change (95% CI)**	***P***	**Heterogeneity**	
					***I*****^2^(%)**	***P***
Gestational age	Cohort study	3	−0.46 (−0.89, −0.04)#	0.032	76.7	0.014
	Non-cohort study	7	−0.24 (−0.44, −0.04)#	0.017	55.3	0.037
prematurity	Cohort study	2	0.62 (0.20, 1.88)^∧^	0.394	76.9	0.038
	Non-cohort study	4	1.08 (0.57, 2.06)^∧^	0.818	77.7	0.004
Birthweight	Cohort study	3	−0.43 (−0.96, 0.10)#	0.115	85.3	0.001
	Non-cohort study	5	−0.34 (−0.72, 0.05)#	0.090	85.0	0.000
Apgar 1	Cohort study	3	−0.64 (−1.00, −0.28)#	0.001	83.8	0.002
	Non-cohort study	7	−0.60 (−0.82 −0.38)#	0.000	48.9	0.068
Apgar 5	Cohort study	3	−0.54 (−0.97, −0.12)#	0.013	88.3	0.000
	Non-cohort study	7	−0.80 (−1.02, −0.58)#	0.000	58.10	0.026
Sepsis	Cohort study	4	1.73 (0.76, 3.96)^∧^	0.191	88.7	0.000
	Non-cohort study	7	2.58 (1.09, 6.13)^∧^	0.032	88.60	0.000
Mechanical ventilation	Cohort study	3	2.38 (1.21, 4.66)^∧^	0.012	56.30	0.101
	Non-cohort study	5	2.36(1.19, 4.68)^∧^	0.014	74.50	0.003

∧ *OR and 95% CI;*

# *SMD and 95% CI*.

*Non-cohort study = case-control study or cross-sectional study*.

**Table 7:** Subgroup analyses based on the definition of AKI.

**Risk factors**	**Diagnostic criteria**	**Number of trials**	**Net change(95% CI)**	***P***	**Heterogeneity**	
					***I*****^2^(%)**	***P***
Age	KDIGO	3	−0.42 (−0.75, −0.09)#	0.014	49.6	0.137
	Non-KDIGO	2	−0.002 (−0.29, 0.28)#	0.987	0.0	0.896
Gestational age	KDIGO	4	−0.41 (−0.89, 0.08)#	0.098	87.5	0.000
	Non-KDIGO	6	−0.26 (−0.41, −0.12)#	0.000	0.0	0.863
prematurity	KDIGO	2	0.56 (0.25, 1.26)^∧^	0.160	71.6	0.060
	Non-KDIGO	4	1.18 (0.59, 2.36)^∧^	0.650	75.8	0.006
Birthweight	KDIGO	4	−0.46 (−1.04, 0.12)#	0.122	91.3	0.000
	Non-KDIGO	4	−0.28 (−0.55, −0.01)#	0.042	59.8	0.058
Apgar 1	KDIGO	6	−0.55 (−0.77, −0.33)#	0.000	70.2	0.005
	Non-KDIGO	4	−0.72 (−0.95, −0.49)#	0.000	13.7	0.324
Apgar 5	KDIGO	5	−0.54 (−0.86, −0.23)#	0.001	81.7	0.000
	Non-KDIGO	5	−0.91 (−1.10, −0.71)#	0.000	36.4	0.178
Sepsis	KDIGO	8	1.88 (0.99, 3.58)^∧^	0.055	89.8	0.000
	Non-KDIGO	3	3.32 (1.78, 6.20)^∧^	0.000	43.8	0.169
Mechanical ventilation	KDIGO	2	2.65 (1.36, 5.19)^∧^	0.004	0.0	0.613
	Non-KDIGO	6	2.28 (1.30, 3.98)^∧^	0.004	75.8	0.001

∧ *OR and 95% CI;*

# *SMD and 95% CI*.

*AKI, acute kidney injury; Non-KDIGO, arbitrary or acute kidney injury network definition; KDIGO, kidney disease: improving global outcomes or kidney disease: improving global outcomes definition modified for neonates*.

### Publication Bias

Assessment of publication bias using Egger's tests showed that there was no potential publication bias among the included trials in the study except for 1-min Apgar score and sepsis (Table 5). However, the results were stable after non-parametric clipping for 1-min Apgar score and sepsis.

## Discussion

This study revealed that early gestational age and low birthweight were significantly associated with an increased risk of AKI in critically ill neonates. This finding is consistent with the review published by Perico et al. (9). This may be attributed to the fact that the earlier the gestational age and (or) lower the birthweight, the lower the number of nephrons and their maturity (32, 33), which leads to an increased susceptibility toward kidney injury (34). However, we found a significant association of AKI with lower gestational age but not with preterm birth gestation (<37 weeks) (35). It is possible that AKI is associated with a lower gestational age cutoff and should be evaluated.

In this study, we observed that CHD may increase the risk of AKI in neonates by nearly three times, which may be due to the decreased renal perfusion induced by unstable hemodynamics (36). We were able to show that hyperbilirubinemia was significantly associated with an increased risk of AKI in critically ill neonates. Possible pathophysiological mechanisms are as follows: (1) Circulatory disturbance caused by liver dysfunction and portal hypertension can lead to renal hypoperfusion; (2) an afferent arterial vasoconstriction caused by inadequate effective circulatory volume and renin–angiotensin–aldosterone activation; and (3) the formation of intratubular bile casts and the direct bilirubin tubular toxicity (37). In agreement with the findings of Nillsen et al., our findings indicated that the risk of AKI in neonates with NEC increased approximately by six times. This may be attributed to the fact that a significant inflammatory cascade caused by NEC can lead to microcirculatory disturbance, resulting in progressive afferent arteriolar constriction and increased pressure within the renal tubules, in turn, producing a sustained loss of filtration (38).

In agreement with the findings of van den et al. regarding AKI in critically ill neonates (39), mechanical ventilation was a risk factor. The study by Koyner et al. elaborated on the possible mechanisms, which all ultimately lead to AKI by decreasing renal perfusion. The specific mechanisms are as follows: (1) The increase in intrathoracic pressure caused by mechanical ventilation can reduce cardiac output by compressing the mediastinal structures and pulmonary vasculature to increase the right ventricular afterload and to decrease the venous return to the heart. (2) Mechanical ventilation can alter a variety of neurohormonal systems including sympathetic outflow, the renin–angiotensin axis, nonosmotic vasopressin release, and atrial natriuretic peptide production. (3) The increased intrathoracic pressure caused by mechanical ventilation has been shown that it may directly correlate with a decrease in renal perfusion and glomerular filtration rate (40).

Constance et al. (41) in their propensity-matched cohort study, observed that combined use of vancomycin in addition to gentamicin did not increase the risk of AKI in neonates. This is similar to our result. However, some studies believe that the use of vancomycin can significantly increase the risk of AKI in children and adults, especially when combined with other nephrotoxic drugs and (or) diuretics (42, 43). Due to the inclusion of fewer studies and the lack of analysis of different doses and treatment courses of vancomycin, the result of this study needs larger sample studies to confirm.

Subgroup analysis based on the research method showed that sepsis was significantly associated with AKI in the non-cohort studies while not significant in the cohort studies. So research method was one of the sources of heterogeneities. The possible explanation is that different types of studies have different strengths of evidence. According to *A Manual for Evidence-based Practice* (44), the exposure data of the cohort studies are collected before the outcome, so the data are reliable and the evidence of causality is good. Case-control studies are easily affected by confounding factors, while it is difficult for cross-sectional studies to determine the order of “exposure” and “outcome.” Therefore, the strength of evidence in case-control studies is inferior to cohort studies, which in cross-sectional studies is even more inferior.

Subgroup analyses based on the definition of AKI showed that age, birthweight, and sepsis had different results between KDIGO-defined and non-KDIGO-defined studies. Meanwhile, some of the heterogeneities have declined after subgroup analyses. So the definition of AKI was one of the sources of meta-analysis. Nowadays, the diagnosis of neonatal AKI has not been unified. There are five definitions that describe the state of neonatal AKI in our meta-analysis: (1) arbitrary definition mainly based on absolute serum creatinine (SCr) ≥1.5 mg/dl;(4, 23, 24, 27), (2) arbitrary definition based on absolute SCr >1 mg/dl and >1.3 mg/dl (for ≥33 weeks and <33 weeks, respectively) after 48 h of life;(12, 22), (3) AKIN definition based on absolute SCr ≥ 0.3mg/dl or SCr ≥1.5 times baseline within 48 h or urine volume <0.5 ml/kg/h for 6 h;(11, 21, 25), (4) KDIGO definition based on absolute SCr ≥0.3 mg/dl within 48 h or SCr ≥1.5 times baseline, which is known or presumed within 7 days, or urine volume <0.5 ml/kg/h for 6 h;(1, 6, 29), and (5) modified KDIGO definition changes the baseline to previous trough value in SCr (2, 5, 20, 26, 28, 30, 31, 45). As we can see, the arbitrary definitions are mainly dependent on an absolute increase in SCr for at least 1 mg/dl, whose critical value is higher than that of AKIN and KDIGO. These definitions do not account for the significance in a percentage increase in SCr and a percentage decrease in urine output. Meanwhile, it is not difficult to find that on the basis of AKIN, KDIGO extended the time to 7 days for percentage increase in SCr. Since the baseline level of SCr changes constantly during the first week of birth, the modified KDIGO definition seems to be more suitable for the diagnosis of neonatal AKI (46). As mentioned above, the KDIGO definitions are more sensitive than AKIN and arbitrary definitions, which may be the reason why the definition of AKI became a source of heterogeneity.

Therefore, subgroup analyses indicated that the results of age, birthweight, and sepsis were not robust. It is necessary to carry out cohort studies to analyze the relationship between risk factors and different stages of AKI in critically ill neonates.

## Limitations

First, this analysis was based on cross-sectional, cohort, and case-control studies, whose controls were not community based, so well-designed multicentric cohort studies are needed to explore the above risk factors that are relied on as causal factors associated with AKI in critically ill neonates. Second, some of the risk factors studied, such as antepartum hemorrhage, hyperbilirubinemia, and vancomycin, were assessed in only two publications, which prevented more robust meta-analyses of these factors. Third, birth asphyxia was not included in this analysis for only one study that provided corresponding data (4). Fourth, among the 17 included studies, only 10 studies excluded congenital anomalies of the kidney and urinary tract (2, 5, 6, 22, 24, 26, 28–31), and 2 studies excluded lethal chromosomal anomaly (2, 5), which may bring some bias to the results. Finally, the studies included have a large time span, and different clinical factors, such as different treatment methods, may bring some bias.

## Conclusions

In this study, we found the incidence of AKI fluctuates from 8.4 to 63.3%. Gestational age, birthweight, 1-min Apgar score, 5-min Apgar score, CHD, hyperbilirubinemia, NEC, sepsis, and mechanical ventilation were risk factors for AKI in critically ill neonates. Well-designed studies with a considerable number of critically ill neonates are necessary to determine the possible link between these nine risk factors and AKI.

## Data Availability Statement

The original contributions presented in the study are included in the article/Supplementary Material, further inquiries can be directed to the corresponding author/s.

## Author Contributions

QH, S-JL, and MW contributed to the study concept and design, article selection and quality assessment, data analysis and interpretation, and manuscript writing. Q-LC, HC, and QL contributed to the study concept and design, and manuscript writing. All authors contributed to the article and approved the submitted version.

## Conflict of Interest

The authors declare that the research was conducted in the absence of any commercial or financial relationships that could be construed as a potential conflict of interest.
