# Supplementary material for: Risk Factors for Acute Kidney Injury in Critically Ill Neonates: A Systematic Review and Meta-Analysis
Source: Front Pediatr. 2021 Jul 14;9:666507. doi: 10.3389/fped.2021.666507 (PMC8316634; doi:10.3389/fped.2021.666507)
Supplement: Supplementary file 1 [file Data_Sheet_1.PDF]

PubMed:

#1 (((((risk) OR risks) OR relative risk) OR relative risks) OR risk, relative) OR risks, relative

#2 (((risk factor[MeSH Terms]) OR risk factor*) OR factor*, risk) OR population* at risk

#3 ((((((Infant, Newborn [Mesh]) OR infant [Mesh]) OR newborn*[Title/Abstract]) OR newborn infant*[Title/Abstract]) OR neonate*[Title/Abstract]) OR infant*, newborn[Title/Abstract]) OR infant*[Title/Abstract]

#4 ((((((acute kidney injury [Mesh]) OR acute kidney injur*[Title/Abstract]) OR acute kidney failure[Title/Abstract]) OR acute kidney insufficienc*[Title/Abstract]) OR acute renal injur*[Title/Abstract]) OR acute renal failure[Title/Abstract]) OR acute renal insufficienc*[Title/Abstract]

#5 #1 OR #2
#6 #3 AND #4 AND #5

Embase:

#1 'risk factor'/exp OR 'risk factor*'

#2 'risk'/exp OR 'relative risk' OR 'relative risks' OR 'risk hypothesis'

#3 'acute kidney failure'/exp OR 'acute kidney failure':ti OR 'acute kidney injur*':ti OR 'acute kidney insufficienc*':ti OR 'acute renal failure':ti OR 'acute renal insufficienc*':ti OR 'kidney acute failure':ti OR 'kidney failure, acute':ti OR 'kidney insufficienc*, acute':ti OR 'renal insufficienc*, acute':ti

#4 'infant'/exp OR ‘ infant*':ti OR 'newborn'/exp OR 'child*, newborn*':ti OR 'full term infant*':ti OR 'human neonate*':ti OR 'human newborn*':ti OR 'infant*, newborn*':ti OR 'neonate*':ti OR 'neonatus':ti OR 'newborn bab*':ti OR 'newborn child*':ti OR 'newborn infant*':ti OR 'newly born bab*':ti OR 'newly born child*':ti OR 'newly born infant*':ti

#5 #1 OR #2

#6 #3 AND #4 AND #5
